# Comparative Evaluation of the Effectiveness and Efficiency of Computational Methods in the Detection of Asbestos Cement in Hyperspectral Images

**DOI:** 10.3390/ma18153456

**Published:** 2025-07-23

**Authors:** Gabriel Elías Chanchí-Golondrino, Manuel Saba, Manuel Alejandro Ospina-Alarcón

**Affiliations:** Faculty of Engineering, University of Cartagena, Cartagena de Indias 130015, Colombia; gchanchig@unicartagena.edu.co (G.E.C.-G.); mospinaa@unicartagena.edu.co (M.A.O.-A.)

**Keywords:** Amianthus, hyperspectral images, remote sensing, asbestos cement detection, computational methods

## Abstract

Among the existing challenges in the field of hyperspectral imaging, the need to optimize memory usage and computational capacity in material detection methods stands out, given the vast amount of data associated with the hundreds of reflectance bands. In line with this, this article proposes a comparative study on the effectiveness and efficiency of five computational methods for detecting composite material asbestos cement (AC) in hyperspectral images: correlation, spectral differential similarity (SDS), Fourier phase similarity (FPS), area under the curve (AUC), and decision trees (DT). The novelty lies in the comparison between the first four methods, which represent the spectral proximity method and a machine learning method, such as DT. Furthermore, SDS and FPS are novel methods proposed in the present document. Given the accuracy that detection methods based on supervised learning have demonstrated in material identification, the results obtained from the DT model were compared with the percentage of AC detected in a hyperspectral image of the Manga neighborhood in the city of Cartagena by the other four methods. Similarly, in terms of computational efficiency, a 20 × 20 pixel region with 380 bands was selected for the execution of multiple repetitions of each of the five computational methods considered, in order to obtain the average processing time of each method and the relative efficiency of the methods with respect to the method with the best effectiveness. The decision tree (DT) model achieved the highest classification accuracy at 99.4%, identifying 11.44% of asbestos cement (AC) pixels in the reference image. However, the correlation method, while detecting a lower percentage of AC pixels (9.72%), showed the most accurate visual performance and had no spectral overlap, with a 1.4% separation between AC and non-AC pixels. The SDS method was the most computationally efficient, running 23.85 times faster than the DT model. The proposed methods and results can be applied to other hyperspectral imaging tasks involving material identification in urban environments, especially when balancing accuracy and computational efficiency is essential.

## 1. Introduction

Remote sensing is a discipline focused on acquiring geo-biophysical information about terrestrial surface objects or phenomena from distant platforms, such as satellites or aircraft, utilizing electromagnetic radiation as the primary medium of interaction [1,2,3,4]. Compared to traditional techniques, remote sensing enables non-invasive, simultaneous observation of extensive areas, making it highly valuable for environmental monitoring and natural disaster assessment [5,6,7,8,9]. Despite high initial investment costs—hardware acquisition accounting for 48% to 78% of total project expenses—operational costs are comparatively lower than those of conventional methodologies once the system is established [10,11,12].

Among the most advanced remote sensing techniques, hyperspectral imaging integrates conventional photography with spectroscopy to capture both spatial and spectral information. Each pixel in a hyperspectral image contains a complete reflectance spectrum, forming a data cube that represents the material’s absorption and scattering properties [13,14]. This imaging modality comprises three dimensions: two spatial (x, y) and one spectral (λ), facilitating detailed multivariate analysis of the observed material and its physicochemical characteristics [15,16]. Unlike conventional or multispectral imaging, hyperspectral imaging captures a continuous and extensive range of wavelengths, providing high spectral resolution per pixel, which enhances material classification accuracy [17,18,19,20,21].

Asbestos cement (AC) materials pose significant public health and environmental risks due to their potential to release asbestos fibers when weathered, damaged, or improperly handled during demolition or maintenance activities [22]. Inhalation of these fibers is associated with asbestosis, lung cancer and mesothelioma, among others [23]. Despite regulatory bans in many countries, AC remains widespread, especially in urban areas within developing regions, making its accurate identification and mapping a pressing priority. Hyperspectral imaging offers a critical advantage over traditional multispectral imaging due to its high spectral resolution, enabling precise detection of subtle spectral features unique to asbestos-containing materials. The detection of AC using remote sensing has gained increasing interest over the last decade, with various studies employing hyperspectral imagery to enhance identification accuracy [22,24,25,26,27,28,29,30,31,32,33,34].

A range of algorithms has been applied in these studies, including object-based image analysis (OBIA), spectral feature fitting (SFF), spectral angle mapper (SAM), machine learning methods such as support vector machine (SVM), random forest (RF), and, more recently, deep learning methods such as convolutional neural networks (CNNs) [1]. These methodologies seek to improve classification performance by leveraging spectral and spatial properties, while balancing the trade-offs between spatial resolution and cost.

Hyperspectral imaging has proven particularly effective in asbestos detection, as demonstrated in studies utilizing shortwave infrared (SWIR) hyperspectral data combined with chemometric techniques to classify asbestos fibers embedded in cement matrices. These approaches have successfully identified distinct spectral signatures of asbestos minerals such as amosite, crocidolite, and chrysotile [35,36]. Meanwhile, machine learning models, including CNNs, have been implemented for AC roof detection in urban areas, using both aerial and satellite imagery. These studies have reported high classification accuracy, reinforcing the potential of deep learning in AC identification [28,37,38].

Beyond classification, hyperspectral imaging has also been applied to assess the degradation of AC roofs, a crucial factor in understanding asbestos fiber dispersion and associated health risks [39,40]. By analyzing spectral variations linked to material deterioration, researchers have gained insights into the environmental and public health implications of aging AC surfaces [40]. Additionally, hyperspectral techniques combined with principal component analysis (PCA) and partial least squares discriminant analysis (PLS-DA) have been effectively employed to distinguish asbestos-containing materials from other construction and demolition debris, further expanding their applicability in waste management [41].

The literature exhibits a notable scarcity of studies utilizing hyperspectral images with more than 100–120 spectral bands. The high dimensionality and redundancy inherent in hyperspectral data, where each pixel contains extensive reflectance information across multiple wavelengths, pose significant computational challenges. Processing such large datasets requires efficient memory management and optimized computational resources, an aspect that remains underexplored in current research. One potential solution involves the implementation of partitioning techniques within parallel computing architectures to enhance processing efficiency [42,43].

Furthermore, the widespread application of hyperspectral imaging in environmental monitoring has underscored the computational burden of real-time processing, which remains a critical challenge [44,45]. Although deep learning techniques, particularly convolutional neural networks (CNNs), have demonstrated high efficacy in material identification, they encounter inherent limitations related to computational demands and the availability of robust training datasets [46]. Consequently, hyperspectral image processing methods must not only achieve high classification accuracy but also be optimized to minimize computational costs and ensure adaptability to systems with constrained resources [47,48]. Therefore, the literature highlighted the need for accurate and computationally efficient material detection methods.

The present study aims to evaluate five alternative approaches for identifying asbestos cement (AC) in hyperspectral images of an urban area. Two novel methods—spectral differential similarity (SDS) and Fourier phase similarity (FPS)—were employed alongside two established techniques—correlation and area under the curve (AUC). All four are classified as spectral proximity methods. In addition, a machine learning approach based on decision trees (DT) was also utilized. These methods integrate both mathematical frameworks and machine learning adaptations, applied to a reference hyperspectral image of the Manga neighbourhood in Cartagena de Indias, Colombia, approximately 0.4 square kilometres (725 × 850 pixels, 380 spectral bands). Efficacy was assessed by comparing AC detection percentages and visually analyzing detected areas against field-verified AC locations. Computational efficiency was evaluated by executing each method on a selected 20 × 20 × 380 data cube across multiple iterations to determine average processing times. Implementation and analysis leveraged open-source libraries such as Spectral, NumPy, Matplotlib, and Pandas.

The rest of the article is structured as follows: Section 2 presents a description of the methodological phases that guided the development of this research. Section 3 presents the results obtained in this research, which include the creation of a dataset of AC and other materials’ spectral signatures, the evaluation of the methods with the created dataset for determining the detection thresholds and accuracy in detection in the case of the decision tree method, the deployment of the methods on the complete reference image, and the evaluation of the effectiveness and computational efficiency of each method. Finally, Section 4 presents the conclusions and future work derived from this research.

## 2. Materials and Methods

**Figure 1:**
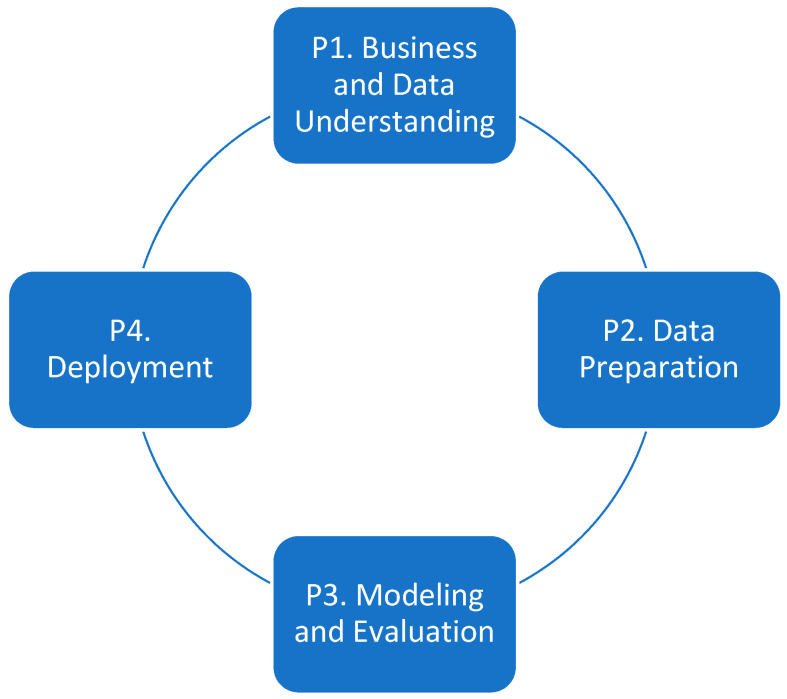
Adaptation of the CRISP-DM Methodology.

For this research, a four-phase adaptation of the CRISP-DM methodology was implemented: Phase 1. Business and Data Understanding, Phase 2. Data Preparation, Phase 3. Modeling and Evaluation, and Phase 4. Model Deployment (see Figure 1). This methodology was selected due to its standardized, flexible, and industry-independent framework, making it adaptable to various data science applications regardless of the technological domain. Its structured approach ensures reproducibility and facilitates implementation across diverse research contexts, including hyperspectral image analysis for AC detection [49,50,51,52].

### 2.1. Phase 1: Business and Data Understanding

**Figure 2:**
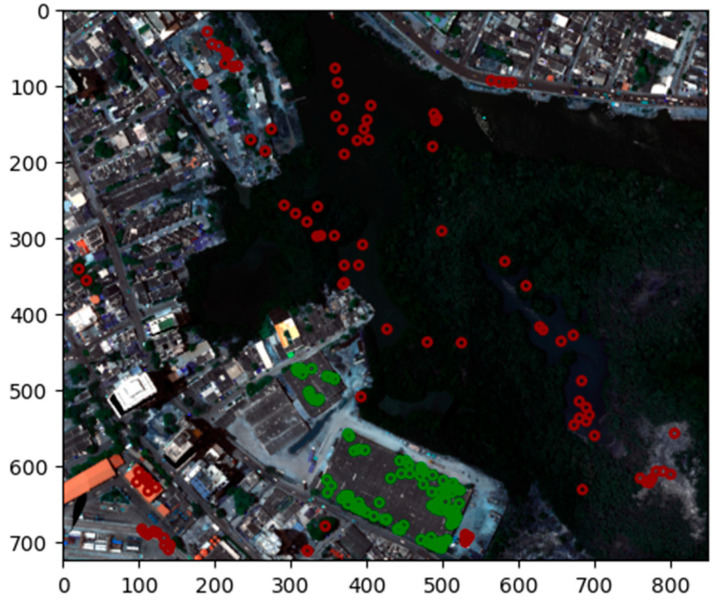
Selected sample pixels of asbestos cement (AC) in green and other materials in red.

**Figure 3:**
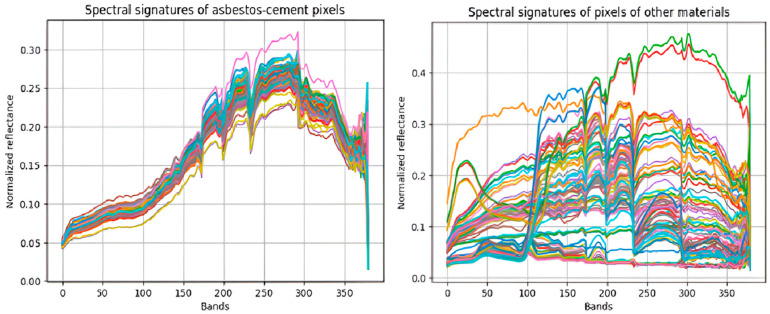
Spectral signatures of asbestos cement (AC) samples and other materials.

In the initial phase of the methodology, a hyperspectral image cube of the Manga neighborhood in Cartagena de Indias, Colombia, was utilized. This reference image, spanning 725 × 850 pixels (totaling 616,250 pixels) with 380 reflectance bands from 400 nm to 2500 nm, served as the foundational source of raw spectral information. From this comprehensive image, a spectral dataset was meticulously constructed. This dataset contains 100 spectral signatures extracted specifically from asbestos cement (AC) pixels. These samples originate from georeferenced locations within the study area, where prior field sampling involved collecting 1 × 1 cm specimens for analysis. Polarized light microscopy (PLM) was then used to examine these specimens, unequivocally confirming the presence of AC [20]. Concurrently, an additional 100 spectral signatures were selected, representing non-asbestos materials (e.g., vegetation, water bodies, metal roofs). These 200 collected spectral signatures constitute the complete spectral dataset for the present study, representing approximately 0.033% of the total pixels in the reference image (Figure 2). As depicted in Figure 3, the sampled AC spectral signatures are highlighted in green, while the spectral signatures of other materials are shown in red, superimposed on the RGB representation of the hyperspectral image.

Each sampled pixel for both asbestos cement (AC) and non-asbestos encompasses 380 reflectance bands, collectively forming a unique characteristic spectral signature. For AC, this signature notably exhibits a set of relevant peaks within bands 150 to 300. Figure 3 illustrates the normalized spectral signatures derived from these sampled pixels. The normalization process was applied uniformly across all 380 reflectance bands. This standardization was performed to enhance computational efficiency and facilitate the calculation of percentage similarities employed by the various detection methods. The normalization method employed involved scaling each reflectance value by subtracting the minimum value of its respective signature and then dividing by the difference between the maximum and minimum values of that same signature. These spectral signatures were obtained using a specialized spectral library, which provides direct access to the reflectance data for each band composing every pixel. Furthermore, the normalization procedure itself leveraged the robust numerical array operation capabilities provided by the NumPy library.

### 2.2. Phase 2: Data Preparation

In the second phase of the methodology, spectral data preparation for both machine learning and non-machine learning approaches was executed. For the non-machine learning methods—correlation, spectral differential similarity (SDS), area under the curve (AUC), and Fourier phase similarity (FPS)—the characteristic spectral signature, or average asbestos cement (AC) pixel, was derived solely from the 100 base AC spectral signatures. The above is based on the fact that these non-machine learning methods use the characteristic spectral signature to compare their similarity with respect to the two groups of sample pixels, obtaining in each case the minimum and maximum similarity percentages, which are then used to determine the reference threshold from which each method begins to detect asbestos cement.

In this regard, the decision tree model utilized the same 200 spectral signatures considered by the other methods, but in this case, they were labeled. Thus, this dataset, comprising 100 asbestos cement (AC) samples labeled as ‘1’ and 100 non-AC samples labeled as ‘0’, was essential. Decision trees, as supervised learning algorithms, require both positive (AC) and negative (non-AC) examples during training to learn distinguishing patterns and establish effective decision boundaries between material classes. As shown in Table 1, this structured dataset contains 381 attributes, where the first 380 correspond to the reflectance bands of the image, and the final attribute serves as the target variable, indicating the class of each signature or pixel.

The characteristic pixel, or representative spectral signature, was established by computing the mean reflectance for each band across the 100 sample asbestos cement pixels, leveraging mathematical functions provided by the NumPy library (version 1.24.2). Concurrently, dataset labeling was executed utilizing the Pandas library (version 1.5.3) in Python 3.10.12. Additionally, for the area under the curve (AUC) and Fourier phase similarity (FPS) methods, this phase involved the extraction of the spectral signature’s area under the curve via the trapezoidal method, and the computation of the fast Fourier transform (FFT) of the characteristic AC spectral signature.

### 2.3. Phase 3: Modeling

While the theoretical foundations of the five evaluated methods (Correlation, SDS, AUC, FPS, and DT) are outlined for completeness, this section prioritizes the implementation choices and parameter configurations specific to the present study. The goal is to highlight the methodological adaptations, threshold tuning strategies, and dataset structuring processes that were uniquely defined to optimize the detection of asbestos cement (AC) in the reference hyperspectral image. All algorithmic implementations were developed using open-source Python libraries (NumPy, SciPy, Pandas, Scikit-learn, Orange, etc.), with attention to reproducibility and adaptability in real-world monitoring contexts.

Where applicable, theoretical formulations have been streamlined in favor of emphasizing dataset preparation, spectral band selection, performance thresholds, and model deployment decisions made during experimentation. For readers interested in the foundational mathematical background of each method, references [53,54] provide additional detail.

**Figure 4:**
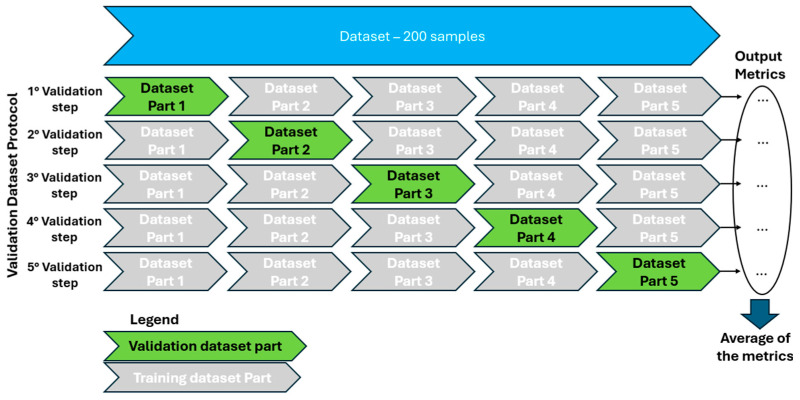
Cross-validation protocol implemented for training and evaluating the decision tree (DT) model.

Thus, in phase 3 of the methodology, the implementation and evaluation of the five detection methods considered were carried out, using the sample spectral signatures for the non-machine learning methods and the spectral signature dataset from Figure 4 for the decision tree model. Thus, for the correlation method, the characteristic pixel is operated by Pearson’s correlation r, which measures the linear relationship between two spectral signatures. It is expressed according to Equation (1) [53].

(1) $$r=\frac{\sum \left(X_{i}-\bar{X})(Y_{i}-\bar{Y}\right)}{\sqrt{\sum {\left(X_{i}-\bar{X}\right)}^{2}\sum {\left(Y_{i}-\bar{Y}\right)}^{2}}}$$

where $X_{i}$ and $Y_{i}$ represent the spectral signatures of two different materials (composed in this case of arrays of 380 bands of normalized reflectance), $\bar{X}$ and $\bar{Y}$ their characteristic pixel. Thus, Equation (1) illustrates the calculation performed with this method between the characteristic pixel and the sample pixels. This pixel is operated individually with the 100 AC pixels and the 100 pixels of other materials, obtaining for each pixel group the minimum and maximum correlation-based similarity percentage. Therefore, if the minimum correlation percentage with AC pixels is not higher than the maximum correlation percentage with other materials, the reflectance band set with no overlap is selected. If no overlap exists, these thresholds are considered when deploying the method on the image.

Instead, with respect to the spectral differential similarity (SDS) method, which is based on calculating the absolute difference between the spectral signature of an unknown material and the reference signatures (Equation (2)), where $X_{i}$ and $Y_{i}$ represent the spectral values in each band [55], the characteristic spectral signature or average pixel is differentiated from the set of AC spectral signatures and the spectral signatures of other materials, obtaining the sum of the differences, so that the closer the difference is to zero, the higher the percentage of similarity between the characteristic spectral signature and the evaluated sample spectral signature.

(2) $$SDS=\sum \left(X_{i}-Y_{i}\right)$$

In this regard, for the two groups of sample pixels, the minimum and maximum similarity percentages were calculated using Equation (2), so that if the minimum similarity percentage with AC pixels is not higher than the maximum similarity percentage with other materials, the reflectance band set with no overlap is selected. If no overlap exists, the determined thresholds are used for deploying the method on the complete image.

Similarly, with respect to the AUC method, the area estimated for the characteristic pixel using the trapezoidal rule, which is a numerical integration technique used to estimate the area under the spectral curve using Equation (3), where $x_{i}$ and $y_{i}$ correspond, respectively, to the band number and the reflectance value of that band in the characteristic spectral signature [56], is differentiated with each of the spectral signatures from the two sample groups, so that the minimum and maximum differences are obtained for each group. If the minimum difference obtained with non-asbestos pixels is not greater than the maximum difference with asbestos pixels, the range of bands with no overlap is selected, and then the new characteristic pixel and its area under the respective curve are determined.

(3) $$AUC=\frac{\sum \left(x_{i+1}-x_{i})(y_{i+1}-y_{i}\right)}{2}$$

Regarding the FPS method, the fast Fourier transform obtained from the spectral signature using Equation (4) is used to determine the Fourier phase similarity with the spectral signatures from the two sample groups, using Equation (5). Thus, for each sample group, the maximum and minimum similarity are obtained, so that if the minimum similarity obtained with AC pixels is not greater than the maximum similarity with pixels of other materials, a new range of bands must be selected, and both the new characteristic pixel and its fast Fourier transform must be calculated. Thus, if the minimum phase similarity obtained with the asbestos cement pixel group is greater than the maximum phase similarity obtained with the pixel group of other materials, the method can clearly differentiate the spectral signature of asbestos cement, and this difference between thresholds can be considered the confidence threshold.

(4) $$X\left(k\right)=\frac{1}{N}\;\sum_{n=0}^{N-1}x(n)\cdot e^{-j\cdot \frac{2\pi }{N}\cdot k\cdot n}$$

The fast Fourier transform (FFT) decomposes a spectral signal into its frequency components. Phase similarity is evaluated by comparing the phases of the transforms, allowing the angular distance between phases to be measured to determine spectral similarity [57]. The FFT converts the original spectral signature into a series of frequency components, from which the phase and magnitude can be extracted. To measure phase similarity, the phases of the transformed spectra of two different materials are compared.

In Equation (4), *X*(*k*) is the result of the Fourier transform at frequency *k*, i.e., the spectral representation of the signal in the frequency domain; *x*(*n*) represents the original signal in the time domain or, in our case, the spectral signature as a function of wavelength; *N* is the total number of points in the signal, i.e., the number of spectral samples; *k* is the frequency index in the transformed domain; *n* represents the spectral band and the negative exponent indicates that a direct transform (from spatial/spectral domain to frequency domain) is being performed [57]. Fourier phase similarity is based on the comparison of the phases of the Fourier transforms of two spectral signatures (Equation (5)). The phase difference between the signatures of two materials $X\left(k\right)$ and $Y\left(k\right)$ is expressed by Equation (6) [58].

(5) $$similarity\left(X(k),Y(k)\right)=\frac{1}{N}\sum_{k}e^{j∆\phi_{k}}\times 100$$

(6) $$\Delta \phi_{k}=\phi X(k)-\phi Y(k)$$

where ϕX(k) is the phase of the Fourier transform of the spectral signature of the material X; ϕY(k) is the phase of the Fourier transform of the spectral signature of the material Y; $∆\phi_{k}$ represent the phases of their respective Fourier transforms. If $∆\phi_{k}\approx 0$ at various frequencies, the spectral signatures exhibit high phase similarity, while large values indicate different materials [57,58].

To evaluate the decision tree (DT) model, the spectral signature dataset (comprising 200 pixels as shown in Table 1) underwent a rigorous cross-validation procedure with five folds. This approach was selected to provide robust performance estimates and mitigate overfitting [59,60], by ensuring the model’s performance was assessed across different training and testing subsets within the same dataset.

Specifically, the 200-pixel dataset was partitioned into five equal parts (Figure 4). In each of the five validation steps, four of these parts (160 pixels: 80 AC and 80 non-AC) were designated as the training set, while the remaining part (40 pixels: 20 AC and 20 non-AC) served as the validation set. This process was iteratively repeated five times, with the validation set systematically rotating across the different partitions. At each iteration, the DT method’s validation metrics were calculated. The final performance metric for the method was then obtained by averaging the metrics from all five validation steps (Figure 4).

The DT model, a supervised classification algorithm, partitions data into subsets based on learned decision rules, constructing decision nodes and terminal leaves to make predictions from a set of features [61]. In this work, a binary classification approach was specifically chosen to optimize computational cost for hyperspectral image analysis, given the capability of such classifiers to accurately detect a specific spectral signature. This strategy has demonstrated effectiveness in various material detection research [62,63]. Node splitting within the DT was based on the minimization of entropy (H) or maximization of information gain (IG), as defined by Equation (7) [54], where S represents the dataset, A is the splitting attribute, and Sv are the resulting subsets. The entire machine learning workflow for the DT model was deployed using the visual programming tool Orange.

(7) $$IG\left(S,A\right)=H\left(S\right)-\sum_{v\epsilon V}\frac{\left|S_{v}\right|}{\left|S\right|}H(S_{v})$$

### 2.4. Phase 4: Model Deployment

In phase 4 of the methodology, the deployment of the methods evaluated in this work was carried out on the complete reference hyperspectral image. For the non-machine learning models, the range of bands where the method is effective and the minimum threshold from which each method begins to differentiate AC were considered. Likewise, for the decision tree model, the fitted model was taken into account, and the prediction of the possible label for each pixel of the image was made. Thus, each of the five methods was applied to the 616,250 pixels of the image, each with 380 bands, identifying the AC pixels. In the case of detecting the material, the pixels were counted and the corresponding areas were colored cyan blue in the RGB representation of the image. For each of the five methods, the percentage of AC was obtained, and the effectiveness of the methods was compared through visual inspection and the similarity in the detected AC pixels.

On the other hand, in this phase, the computational efficiency of the five methods considered was also evaluated by performing multiple repetitions (25, 50, 75, 100) of each method on a 20 × 20 pixel region of the reference image with 380 reflectance bands, using the advantages provided by the timeit library in Python. Based on the repetitions performed, the average time per repetition and the total average time were obtained for each method, thus allowing the identification of the method with the best computational efficiency.

## 3. Results

**Figure 5:**
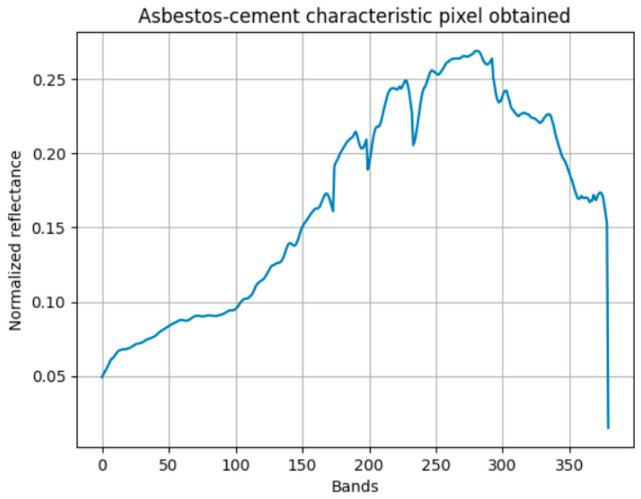
The characteristic spectral signature obtained from them.

The initial phase of this investigation involved the derivation of an average spectral signature representative of composite AC materials, which served as the reference standard for the non-machine learning-based classification algorithms. As depicted in Figure 5, this characteristic spectral signature was generated by calculating the mean reflectance for each spectral band across a cohort of 100 representative pixel samples.

**Figure 6:**
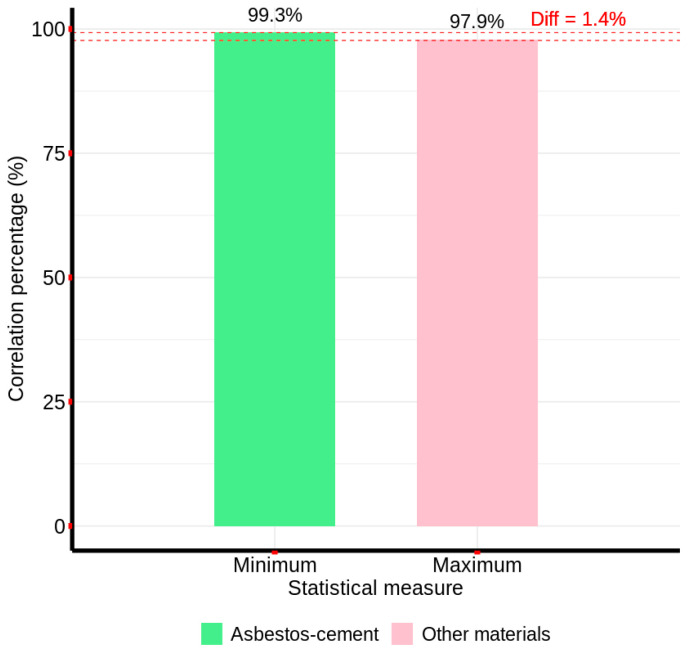
Pearson correlation coefficients between the characteristic spectral signature of AC and the spectral signatures of non-AC materials.

Starting from the characteristic spectral signature presented in Figure 5, the correlation method was implemented and evaluated with the sample spectral signatures of AC and other materials, using the advantages provided by the scipy library, which allows obtaining the Pearson correlation between two numerical arrays—in this case, two reflectance arrays (one array with the reflectance of the bands of the characteristic spectral signature and one array with the reflectance of the bands of each sample pixel). In this way, the aim was to identify the correlation method’s ability to differentiate between the asbestos cement spectral signature and the two groups of sample pixels, such that the higher the correlation of the spectral signature with the asbestos cement sample pixels and the greater the difference with the correlation to the pixels of other materials, the better the method’s capability. Thus, in Figure 6, the minimum and maximum correlation percentages obtained with the two groups of spectral signatures are presented.

Analysis of Figure 6 reveals that the correlation-based method demonstrated a distinct discriminatory capability, wherein the minimum correlation coefficient observed for AC pixels exceeded the maximum correlation coefficient for non-AC pixels by a margin of 1.4%. This separation indicates a lack of spectral overlap between the two material classes across the full spectral range of the image, substantiating the method’s efficacy for unambiguous AC detection. Similarly, the spectral differential similarity (SDS) method, utilizing the characteristic spectral signature derived from Figure 5 as a reference, was evaluated. Employing the numerical computation capabilities of the numpy library in Python, the SDS method calculated the cumulative band-wise difference between the reference spectral signature and each sample pixel’s spectral signature.

**Figure 7:**
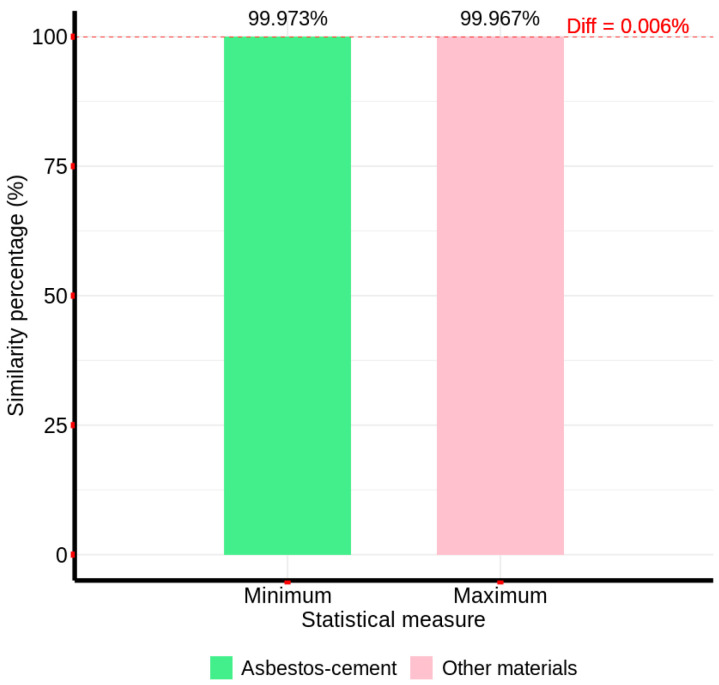
Spectral Differential Similarity values between the characteristic AC spectral signature and the spectral signatures of non-AC sample pixels.

Figure 7 presents the minimum and maximum similarity percentages, derived from these cumulative differences, for both asbestos cement and non-AC pixel groups, illustrating the method’s discriminatory power.

The spectral differential similarity (SDS) method, as depicted in Figure 7, exhibited a discriminatory capacity, albeit with a marginal difference. Specifically, the minimum similarity percentage for AC pixels surpassed the maximum similarity percentage for non-AC pixels by 0.006%. Despite this reduced differential compared to the correlation-based method, the absence of spectral overlap across the image’s full spectral range validates the SDS method’s potential for AC detection.

**Figure 8:**
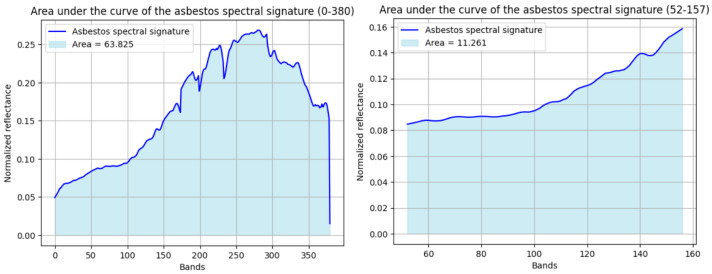
Area under the curve values computed for the characteristic AC spectral signature over two spectral band ranges: 0–380 (entire spectrum) and 52–157 (optimized subrange).

Subsequently, the area under the curve (AUC) method was implemented, utilizing the reference spectral signature from Figure 5. Initial analysis, calculating the AUC across 380 spectral bands, revealed an overlap between asbestos cement and non-AC pixels, indicating inadequate discrimination. Specifically, the maximum difference in AUC for AC pixels was observed to be greater than the minimum difference for non-AC pixels. To mitigate this overlap, an iterative band selection process was conducted. This process identified the spectral range from bands 52 to 157 as exhibiting no overlap. Consequently, Figure 8 presents the AUC values for both the full 380-band range (60.385) and the optimized 52–157 band range (11.261%), demonstrating the impact of spectral band selection on discriminatory performance.

**Figure 9:**
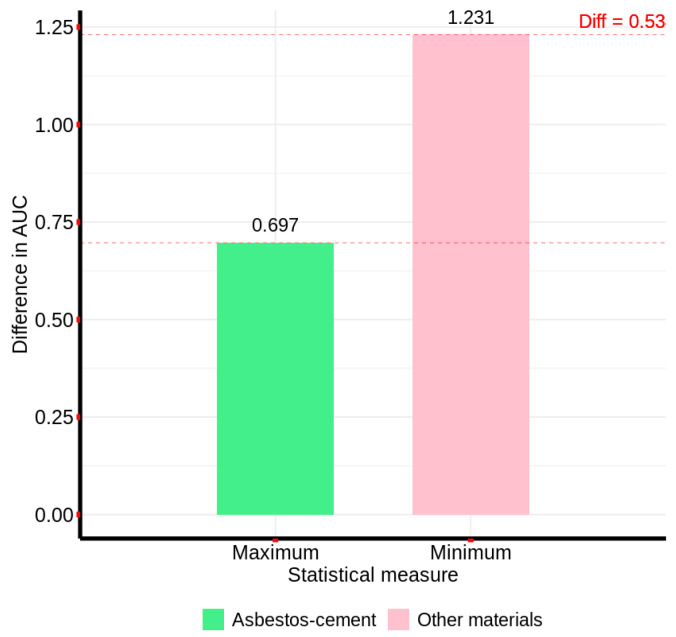
Differences in area under the curve between the characteristic AC signature and sample pixels from non-AC material groups.

Thus, based on the AUC calculated for the band range from 52 to 157, the differences with the areas under the curve for the two groups of pixels or sample signatures were calculated, so that for each group, the minimum and maximum differences were determined, obtaining the results presented in Figure 9.

Figure 9 illustrates that, utilizing the area under the curve (AUC) derived from the characteristic spectral signature within the 52–157 band range, a discernible differentiation was achieved. Specifically, the difference between the minimum AUC for non-AC pixels and the maximum AUC for AC pixels was 0.53, indicating the absence of spectral overlap. Consequently, this method demonstrates applicability for AC detection across the full hyperspectral image.

**Figure 10:**
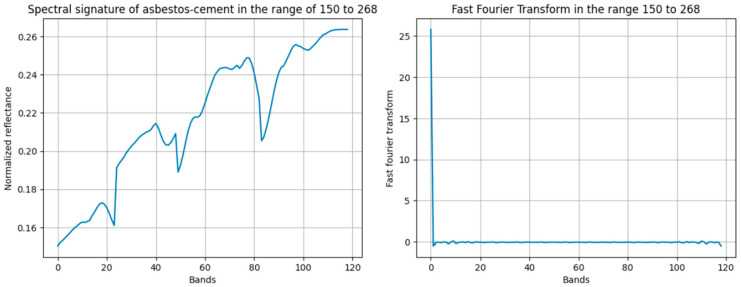
Average pixel and fast Fourier transform obtained for the band range from 150 to 268.

For the Fourier phase similarity (FPS) method, the fast Fourier transform (FFT) of the characteristic spectral signature, as depicted in Figure 5, was computed. This transform was then operated upon the FFT of each pixel from both sample groups, employing the FPS algorithm. Initial analysis, considering all 380 spectral bands, revealed an overlap in phase similarity between asbestos cement and non-AC pixels. To resolve this, an iterative band range optimization was performed. This optimization identified the 150–268 band range as providing adequate differentiation between asbestos cement and non-asbestos cement spectral signatures. Figure 10 presents the modified characteristic pixel, derived from the 150–268 band range, along with its corresponding FFT.

**Figure 11:**
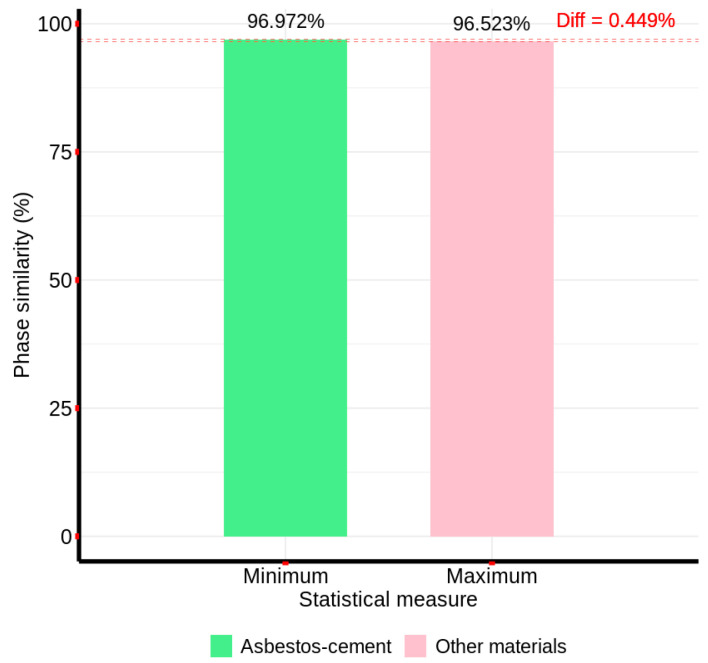
Fourier phase similarity values between the characteristic AC spectral signature and sample pixels from non-AC material groups, calculated over the 150–268 band range.

Now, by finding the FPS between the Fourier representation for the band range from 150 to 268 and the sample pixels from the two groups, the minimum similarity with AC pixels and the maximum similarity with pixels of other materials were obtained, as presented in Figure 11.

According to the results presented in Figure 11, it can be observed that the minimum phase similarity with the sample pixels of AC exceeds the maximum phase similarity with pixels of other materials by 0.449%. Thus, since there is no overlap in the band range from 150 to 268, this method can be applied to the entire reference image.

**Figure 12:**
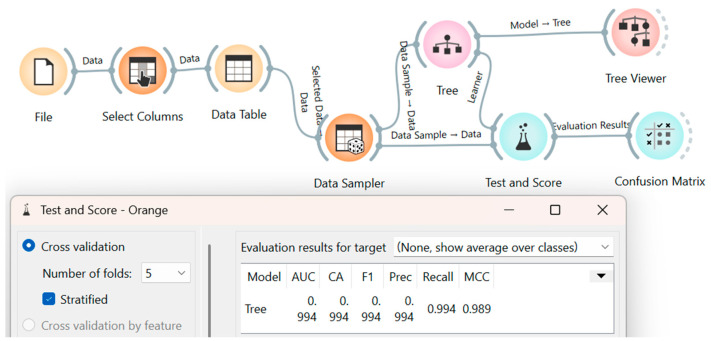
Machine learning workflow for the decision tree model.

Regarding the decision tree (DT) model, the dataset of spectral signatures from Figure 4 was used, and the machine learning workflow was deployed through cross-validation with five folds, using the visual programming tool Orange, which allows for the visual creation, evaluation, and comparison of machine learning models [64,65,66]. Thus, in Figure 12, the diagram with the workflow implemented for the DT model and the results obtained from the performance metrics are presented.

Figure 12 shows the machine learning workflow implemented within the Orange data mining suite. This workflow encompassed several key components: the ‘File’ module for spectral signature dataset ingestion; the ‘Select Columns’ module for predictor attribute specification, specifically the pixel classification label; the ‘Data Sampler’ module for stratified data partitioning utilizing five-fold cross-validation; the ‘Tree’ module for decision tree (DT) model instantiation and hyperparameter optimization; the ‘Tree Viewer’ module for visual representation of the derived decision tree and associated inference rules; the ‘Test Score’ module for performance metric evaluation, including accuracy, precision, recall, and F1-score; and the ‘Confusion Matrix’ module for visualizing classification performance.

**Figure 13:**
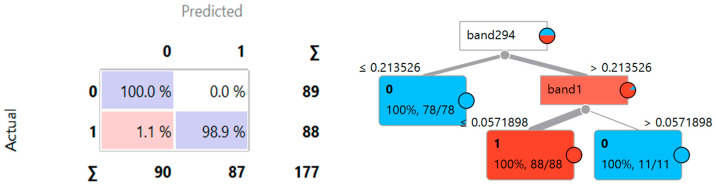
Decision tree and confusion matrix derived from the model.

The classification efficacy of the DT model, as evidenced by Figure 12, demonstrated a high degree of accuracy. Specifically, the metrics for classification accuracy (CA), precision (Prec), and recall were all 0.994, indicating robust model performance in discriminating between asbestos cement and non-AC pixels. Furthermore, an area under the receiver operating characteristic curve (AUC) of 0.994 corroborated the model’s strong discriminative capacity, while an F1-score of 0.994 signified an optimal balance between precision and recall. These results collectively validate the DT model’s generalizability for the balanced 200-signature dataset. This performance is further substantiated by the confusion matrix presented in Figure 13, which illustrates a 100% classification accuracy for label 0 (non-asbestos cement) and a 98.9% accuracy for label 1 (asbestos cement). Additionally, Figure 13 depicts the derived decision tree, highlighting spectral bands 294 and 1 as critical features for AC discrimination.

**Figure 14:**
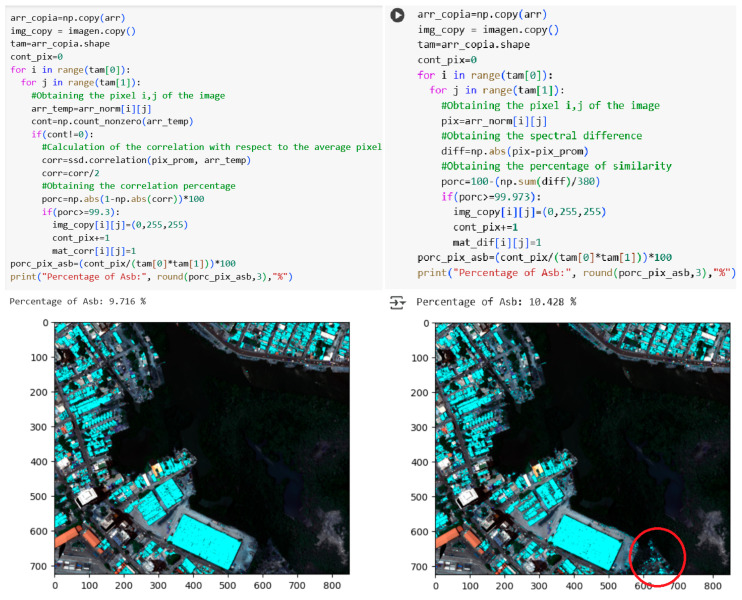
Detection results of the correlation and spectral differential similarity methods applied to the full hyperspectral image of the Manga neighborhood (RGB composite with overlaid detection highlighted in the red circle).

Once the thresholds for the non-machine learning methods and the tuning capability of the decision tree model were identified, the five methods were deployed on the complete reference hyperspectral image. Thus, in Figure 14, both the implementation of the methods and the AC areas detected in cyan blue on the reference image are presented for the correlation and SDS methods.

Comparative analysis of Figure 14 reveals a disparity in AC pixel detection between the correlation and spectral differential similarity (SDS) methods. Specifically, the correlation method identified 9.716% of AC pixels within the reference image, while the SDS method detected 10.428%. However, visual inspection indicates that the SDS method’s output includes extraneous regions potentially representing heterogeneous mixtures rather than pure asbestos cement roofing. Notably, both methods employ pixel-wise iterative calculations to determine similarity metrics, utilizing the scipy library for correlation and the numpy library for SDS.

**Figure 15:**
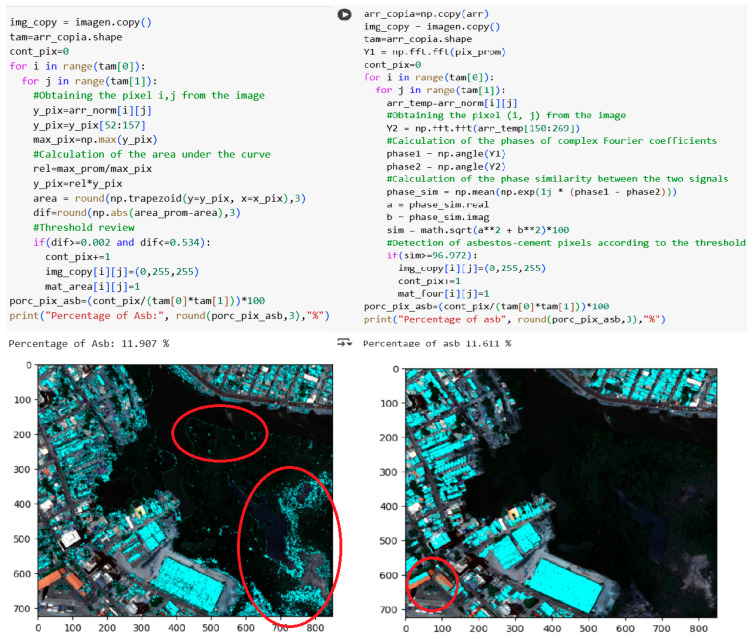
Detection results of the AUC and FPS methods applied to the full hyperspectral image of the Manga neighborhood (RGB composite with cyan blue overlay highlighted in the red circles).

Progressing to the remaining spectral analysis techniques, Figure 15 illustrates the implementation of the area under the curve (AUC) and Fourier phase similarity (FPS) methods, along with the corresponding asbestos cement detection results, delineated in cyan blue, superimposed on the reference image.

On the other hand, Figure 15 presents a comparative analysis of AC pixel detection using the area under the curve (AUC) and Fourier phase similarity (FPS) methods. The AUC method identified 11.907% of AC pixels within the reference image, while the FPS method detected 11.611%. Both methods exhibited classification errors; specifically, the AUC method misclassified vegetation and water regions as AC pixels, whereas the FPS method, while exhibiting results similar to the correlation method, misclassified certain metal roofing materials. Notably, the AUC method demonstrated the highest incidence of visual classification errors among the four non-machine learning methodologies. Both AUC and FPS methods employed pixel-wise iterative calculations, utilizing functionalities provided by the numpy library for AUC computation and fast Fourier transform (FFT) operations.

**Figure 16:**
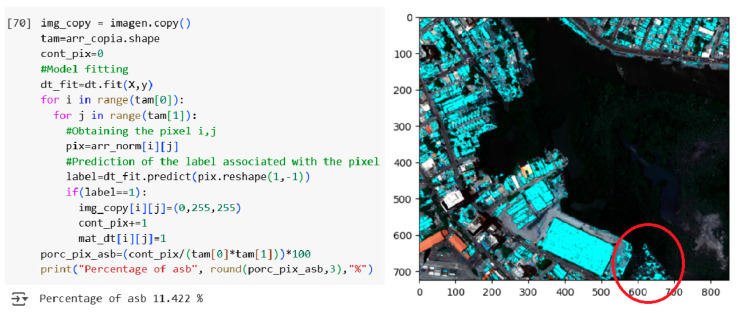
Detection results of the decision tree (DT) model applied to the full hyperspectral image of the Manga neighborhood (RGB composite with cyan blue overlay highlighted in the red circle).

Moving to the decision tree (DT) model, Figure 16 illustrates its implementation using the scikit-learn library in Python, along with the corresponding asbestos cement detection results, delineated in cyan blue, superimposed on the reference image.

**Figure 17:**
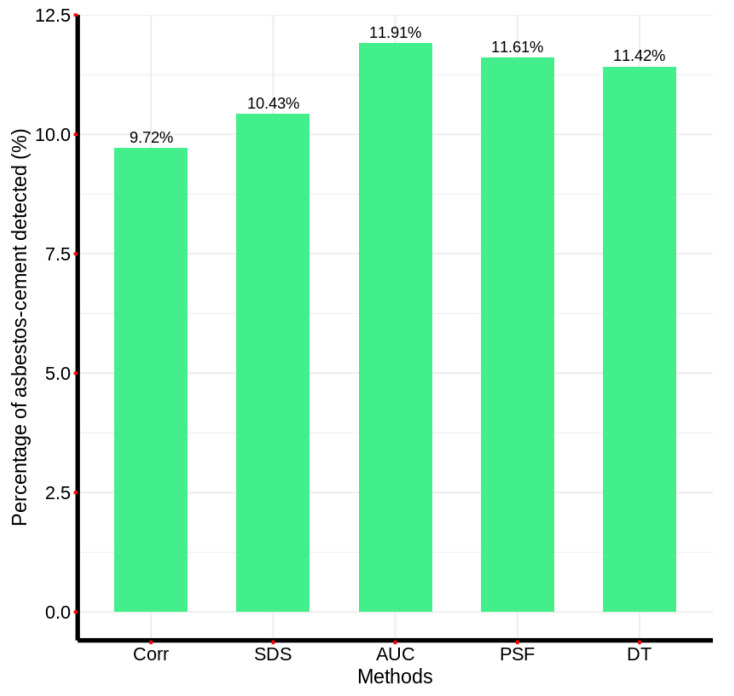
Comparative analysis of the percentage of AC pixels detected by each of the five evaluated computational methods.

Figure 16 demonstrates that the decision tree (DT) model identified 11.442% of AC pixels within the reference image. It is worth mentioning that this percentage refers to the proportion of pixels in the image that were detected as asbestos cement and differs from the 98.9% presented in Figure 13, which corresponds to the accuracy rate of the decision tree method in differentiating asbestos cement pixels from those of other materials. In contrast to the correlation method, which exhibited the fewest visual classification errors, the DT model, akin to the spectral differential similarity (SDS) method, detected extraneous regions not exclusively composed of asbestos cement roofing, potentially indicating areas with partial spectral similarity. To synthesize the detection performance of each method, Figure 17 presents a comparative bar chart illustrating the percentage of AC pixels identified by the five evaluated methodologies.

Furthermore, illustrates a comparative analysis of AC pixel detection percentages across the evaluated methodologies. Notably, the correlation method, which also exhibited the most accurate visual detection, identified the lowest percentage of AC pixels, at 9.72%. Conversely, the area under the curve (AUC) method, characterized by the highest incidence of visual classification errors, detected the highest percentage of AC pixels, at 11.91%.

**Figure 18:**
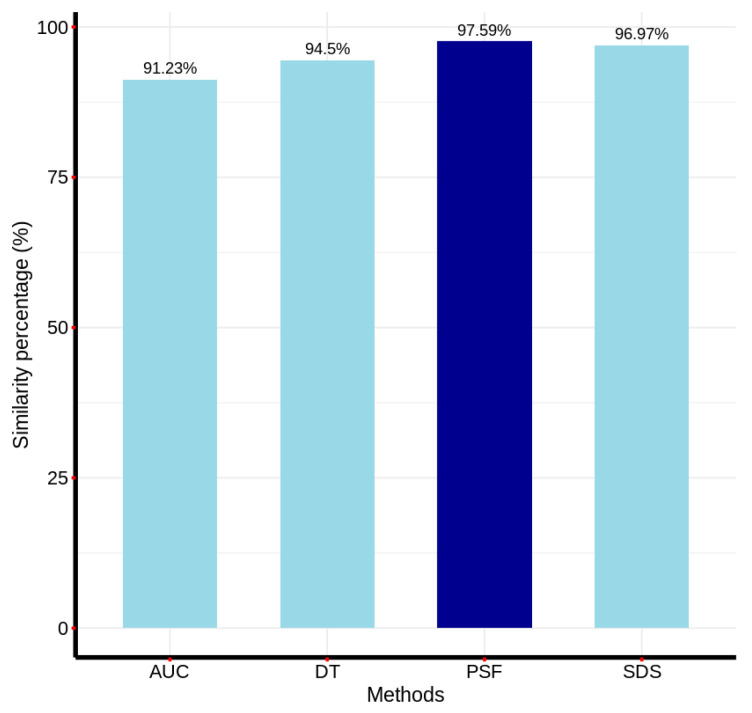
Pixel-wise similarity between the AC detection results of each method and those of the correlation method, used as a reference.

To quantitatively assess the agreement between the remaining detection methods and the visually validated correlation method, a pixel-wise matching analysis was performed. This analysis involved the generation of binary detection matrices, congruent in size with the reference image, for each method. In these matrices, a value of ‘1’ denoted AC pixel identification, while ‘0’ represented non-asbestos cement classification. Figure 18 presents the resulting matching percentages for each method relative to the correlation method, providing a quantitative measure of inter-method consistency.

Figure 18 illustrates the inter-method consistency in AC pixel identification, revealing that the Fourier phase similarity (FPS) method exhibited the highest degree of agreement with the correlation method, followed by the spectral differential similarity (SDS) method. Conversely, the area under the curve (AUC) and decision tree (DT) methods demonstrated the lowest concordance in detected pixels relative to the correlation method. To evaluate the computational efficiency of the five methodologies, a series of iterative executions (25, 50, 75, 100 repetitions) was conducted on a localized 20 × 20 pixel region of the reference 380-band hyperspectral image. This approach mitigated the computational overhead associated with full-image processing. The Python timeit library was employed to measure the total execution time for each method, facilitating a quantitative assessment of processing speed.

**Figure 19:**
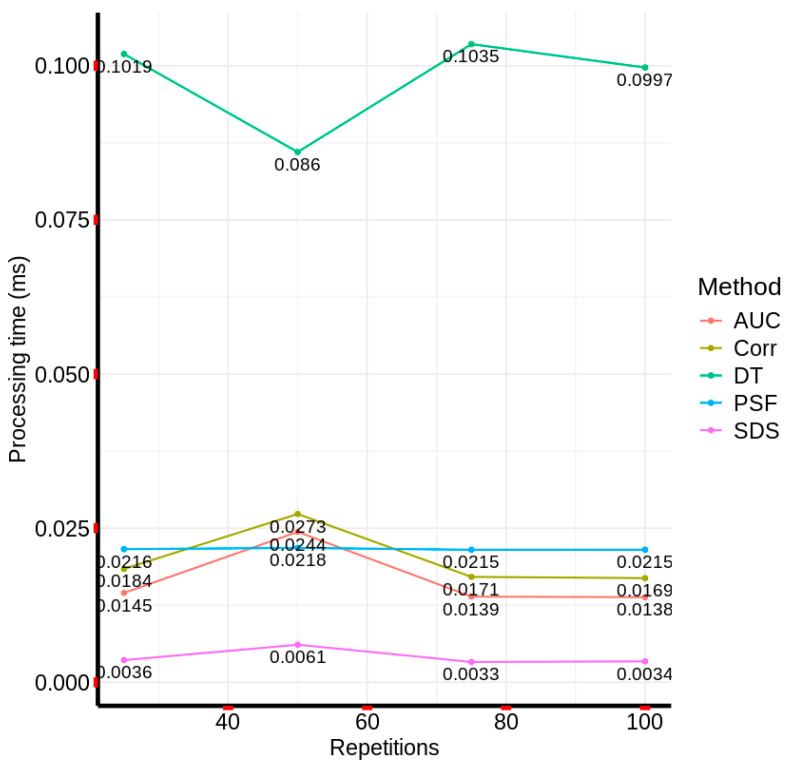
Average computational processing time (in milliseconds) for each method across multiple repetitions on a 20×20 pixel region of the hyperspectral image (380 bands).

Figure 19 presents a graphical comparison of the average processing times for each method across the varying repetition counts. The results depicted in Figure 19 indicate that the SDS method exhibited the highest computational efficiency, while the DT method demonstrated the lowest, with execution times consistently exceeding 0.085 milliseconds across all repetitions. Although the correlation, AUC, and FPS methods exhibited comparable processing times, the FPS method displayed the most consistent and optimal performance. This consistency of the FPS method is clearly observed in Figure 19, where the curve representing the method’s behavior across different repetitions is the one that most closely resembles a straight, or nearly constant, line, with a maximum value of 0.0218 ms and a minimum value of 0.0215 ms, such that the maximum difference is merely 0.0003 ms. Comparative analysis of the most efficient methods (correlation and SDS) against the DT method, based on total average execution time, revealed that the correlation and SDS methods were, on average, 4.92 and 23.85 times faster, respectively. This disparity underscores the superior suitability of correlation and SDS methods for real-time monitoring applications, particularly when contrasted with computationally intensive machine learning models such as decision trees.

## 4. Discussion

It is worth highlighting that, in this work, five computational methods for asbestos cement detection in hyperspectral images were compared, with four of them being non-machine learning-based methods and the decision tree method. Although all methods presented significant results with the pixels belonging to the two sample groups, when deploying the five methods on the complete reference image, visual inspection showed that the correlation method had the fewest classification errors for asbestos cement, while the AUC method showed the greatest confusion in detecting this material. Additionally, although the SDS, FPS, and DT methods detected common areas with the correlation method, they also detected other materials that have a similar spectral signature but do not correspond to the material used in asbestos cement roofs. In this regard, it was observed that the method detecting the largest number of similar pixels was the FPS method, making it highly important for future research to adjust or hybridize this method in order to expand the difference between the minimum phase similarity with AC pixels and pixels of other materials. Furthermore, although it was expected that the decision tree method would be more effective in detecting asbestos cement, this method did not fully detect the spectral signature of the asbestos cement roof material as the correlation model did. Therefore, it is concluded that such models could improve their effectiveness in detecting the shape of signatures if the dataset could discriminate between the material variants with different labels. In this sense, this research contributes to the evaluation and refinement of detection effectiveness in studies such as those mentioned in the literature [67,68], where good effectiveness was achieved using decision tree models and other models for detecting materials with hyperspectral images.

On the other hand, based on the challenges related to computational efficiency posed by the use of hyperspectral images in real-time image monitoring [44,69], this paper found that the correlation and SDS methods were 4.92 times and 23.85 times more efficient in processing a 20 × 20 pixel hyperspectral image with 380 bands compared to the decision tree model. Thus, based on the results obtained, these methods can be highly useful in scenarios where real-time material identification is needed on images with large coverage, such as in environmental monitoring. Additionally, although deep learning-based methods may be more effective [70,71], the correlation and SDS methods can provide a good approximation in detection, with good computational efficiency. Furthermore, given their simplicity in implementation compared to more complex approaches, they may be easier to hybridize and integrate into parallel computing architectures for real-time hyperspectral image analysis. It is worth mentioning that the correlation and SDS methods are based on band-by-band comparison and may not capture the nonlinear relationships in hyperspectral data.

This research stands out for the use and leveraging of open-source domain libraries and technologies in hyperspectral image preprocessing, as well as the implementation, evaluation, and deployment of material detection methods in these images. This is a key contribution for educational institutions and research centers to experiment, replicate, and extrapolate these methods for detecting different types of materials. In this regard, these tools offer a competitive alternative to proprietary tools that have been used in material detection in hyperspectral images [72,73], with the advantage of customization and hybridization capabilities for detection methods.

The novelty of the present study is highlighted in Table 2, where research was carried out with Scopus in July 2025, using the following string as a prompt: “Method” AND “Hyperspectral”, with the results reported in column “All Materials”, while in the AC column, the string prompt results are reported for the string prompt “Method” AND “Asbestos” AND “Hyperspectral” within the “Article title, Abstract, Keywords” search criteria.

Table 2 shows how the methods implemented in the present study are well-known in the literature and are used in a number of cases and fields in hyperspectral images. Nevertheless, they are not commonly used for AC detection. Some applications are found for the DT method.

Compared to previous studies, the present research makes a distinctive contribution by prioritizing not only detection accuracy but also computational efficiency in the identification of asbestos cement (AC) using hyperspectral imagery. The methods developed here are particularly valuable because they can be applied in a broader range of contexts, including in developing countries where AC has recently been banned and where resources for more complex or expensive software solutions may be limited.

Conducting regular AC inventories in urban areas is essential to track the progress of removal efforts and to maintain an up-to-date understanding of the distribution of AC materials. Such monitoring can support local authorities in planning more effective removal strategies and implementing targeted environmental controls, thereby reducing the risks associated with illegal dumping of AC tiles and contributing to improved public health and environmental protection.

While the study by [38] focused on the use of convolutional neural networks (CNNs) applied to RGB and CIR aerial imagery and achieved a high producer’s accuracy (up to 89%), it required detailed training data and complex model training procedures, which may be computationally expensive and less adaptable in resource-limited settings. In contrast, the present study explores non-learning-based techniques like spectral correlation and AUC, which do not require prior training. We found that simpler methods like SDS and correlation not only offer competitive detection performance (with visual concordance rates above 96%) but also outperform decision trees in terms of processing time (being up to 23.85 times more efficient). This positions our approach as a more scalable and lightweight alternative, especially useful in large-scale urban scenarios where computational resources are a constraint.

Moreover, while the study by [31] applied supervised learning techniques to classify the deterioration state of AC roofs using selected hyperspectral bands, their emphasis was on band selection and deterioration condition assessment rather than initial material detection. In contrast, this study maintains a broader urban material identification focus without limiting it to a specific spectrum or requiring large ground-truth datasets. It also highlights the adaptability of open-source tools for implementation, allowing for easier replication and adoption across regions. In this way, the present research fills a methodological gap by offering a comparative framework that balances accuracy and efficiency, supporting decision-makers and researchers in selecting appropriate detection strategies depending on computational capacity and operational goals.

Regarding scalability, the correlation and SDS methods proved highly scalable due to significantly lower computational requirements; they were, on average, 4.9 and 23 times faster than the DT model. This makes them well-suited for processing large hyperspectral datasets typical in environmental monitoring. In contrast, while the DT model achieved high accuracy (up to 98.9%), its computational cost limits scalability without parallel computing optimization, which we propose as future work. On the other hand, in terms of detection accuracy, all methods identified AC pixels within a narrow range. The correlation method exhibited the fewest visual errors, whereas the AUC method, despite detecting the highest AC percentage, misclassified vegetation and water pixels. Pixel concordance analysis showed that the FPS method achieved the closest match (97.59%) to the correlation method, indicating strong reliability for AC detection.

Open-source tools enabled flexibility and cost-effectiveness, allowing adaptation for educational and research purposes. However, certain constraints exist, such as limitations in handling very large datasets without custom optimization or parallel processing, which the authors aim to address in future implementations. Furthermore, two main constraints were identified: potential bias from a relatively limited dataset of AC and non-AC samples, which may not fully capture spectral variability, particularly regarding different asbestos fiber types or concentrations; and possible performance limitations inherent to open-source tools when managing large-scale operational deployments compared to proprietary solutions. Future work will address these limitations by expanding the dataset, refining thresholds for spectral methods, and exploring deep learning models and parallel computing architectures to improve both accuracy and efficiency.

## 5. Conclusions

This paper proposed a comparison of the effectiveness in AC detection and computational efficiency of five different methods. In terms of detection effectiveness, it was determined that the evaluated methods detected AC percentages in the reference hyperspectral image ranging from 9.72% to 11.91%, with the correlation method detecting the least AC and exhibiting the fewest visual inspection errors, while the AUC method detected the most AC pixels but showed a higher number of inspection errors by confusing some vegetation and water pixels with AC pixels. Similarly, when comparing the match in the pixels detected by the correlation method with the four remaining methods, matrix comparison operations were performed, showing that the FPS method had the highest concordance in the detected points, with 97.59%, followed by the SDS method with a concordance percentage of 96.97%. In this regard, although the use of machine learning models in detection is highlighted in the state of the art, the decision tree model ranked third in terms of pixel concordance compared to the correlation method. One possible explanation for this is that the model may require specific labels for different types of materials, while the other methods seem to better detect the shape of the curve. Thus, both the correlation model and the SDS and FPS models can be considered valid alternatives for material detection in the context of hyperspectral images.

Regarding the evaluation of computational efficiency, this research performed multiple repetitions (25, 50, 75, and 100) of the five evaluated methods, finding that the method with the best processing time across the different repetitions was the SDS, while the DT-based method had the highest times in the different repetitions. Therefore, the correlation and SDS methods were, on average, 4.92 and 23.85 times faster in processing the reference hyperspectral image. These results are significant, considering that in scenarios like environmental monitoring, one of the key requirements is computational efficiency due to the size and extensive coverage of the images.

A notable aspect of this research is that, for the preparation of the images, as well as the implementation, evaluation, and deployment of the five methods, open-source domain tools and technologies were used. This work can be adapted, replicated, and extrapolated by educational institutions and research centers for experimentation with hyperspectral images, offering a competitive alternative to proprietary tools in terms of cost, customization, and hybridization of detection methods.

As future work derived from this research, it is proposed, first, to implement and comparatively evaluate the effectiveness and efficiency of deep learning-based machine learning models for AC detection in hyperspectral images. Additionally, considering that the FPS method had the best match with the pixels detected by the correlation method and showed more consistent results in computational efficiency, the aim is to optimize the detection thresholds so that the AC spectral signature can be detected more effectively. Finally, we intend to deploy these methods on an architecture supported by parallel computing. A limitation of this study was the unknown impact on the spectral signature of the percentage and type of asbestos fibers in the AC samples, as well as the relatively limited number of AC and non-AC samples used for training and validation, which should be improved in future research.

A limitation of the current study is the lack of spatial or temporal cross-validation due to the use of a single hyperspectral image and constrained field data. Future work will focus on acquiring multi-temporal or multi-location datasets to assess and improve the generalizability of the detection models.

## Figures and Tables

**Table 1:** Labeled dataset of normalized spectral signatures used for asbestos cement (AC) detection.

Band 1	Band 2	Band 3	Band 4	Band 5	Band 6		Band 376	Band 377	Band 378	Band 379	Band 380	Pixel
0.054957	0.057452	0.059094	0.060998	0.062640	0.064544	…	0.129744	0.124032	0.123606	0.125870	0.015102	1.0
0.046750	0.048817	0.049705	0.051477	0.053185	0.055206	…	0.149179	0.143664	0.134603	0.125073	0.015102	1.0
0.048326	0.048967	0.051149	0.052331	0.053316	0.054695	…	0.181404	0.164741	0.143401	0.119107	0.015102	1.0
0.051018	0.052988	0.054448	0.055546	0.056927	0.058437	…	0.175772	0.162640	0.147538	0.133749	0.015102	1.0
0.050069	0.052200	0.053710	0.055351	0.057124	0.059291	…	0.202304	0.205581	0.210768	0.216087	0.015102	1.0
0.026200	0.030007	0.030860	0.031976	0.032830	0.033615	…	0.056027	0.054038	0.049442	0.040381	0.015102	1.0
…	…	…	…	…	…	…	…	…	…	…	…	…
0.030860	0.030794	0.030860	0.030926	0.031057	0.031385	…	0.053551	0.056437	0.060341	0.062049	0.015102	0
0.025148	0.026067	0.026327	0.027577	0.028431	0.029350	…	0.064675	0.076034	0.086850	0.101773	0.015102	0
0.096535	0.100197	0.104728	0.109652	0.114905	0.122062	…	0.213198	0.196717	0.178861	0.161786	0.015102	0
0.024097	0.024425	0.025082	0.025542	0.026330	0.027249	…	0.056730	0.061129	0.060144	0.058043	0.015102	0

Note: Each row represents a sample pixel described by its reflectance values across 380 spectral bands (Band 1 to Band 380), followed by the class label in the last column (“Pixel”), where 1 indicates an asbestos cement (AC) sample and 0 a non-AC sample (e.g., vegetation, water, metal roofs). All reflectance values were normalized using min–max scaling.

**Table 2:** Summary of literature on computational methods applied to hyperspectral image analysis, with emphasis on AC detection.

Method	All Materials	AC	Reference
Correlation	484	-	-
Spectral Differential Similarity (SDS)	-	-	-
Fourier Phase Similarity (FPS)	-	-	-
Area Under The Curve (AUC)	129	-	-
Decision Tree (DT)	1347	3	AC [25,31,38]
Total	1960	3	-

Note: The “All materials” column indicates the number of articles retrieved in Scopus (as of July 2025) using the search term “Method” AND “Hyperspectral”. The “AC” column reflects the number of results specifically related to asbestos cement, based on the search “Method” AND “Asbestos” AND “Hyperspectral” within article titles, abstracts, and keywords. This review highlights the research gap regarding the application of certain methods (e.g., SDS, FPS) to AC detection.

## Data Availability

The datasets presented in this article are not readily available because the data are part of an ongoing study. Requests to access the datasets should be directed to professor Manuel Saba.

## Abbreviations

The following abbreviations are used in this manuscript:

AC	Asbestos Cement
AUC	Area Under the Curve
SDS	Spectral Differential Similarity
FPS	Fourier Phase Similarity
